# Increased cardiovascular mortality in females with the a/a genotype of the SNPs rs1478604 and rs2228262 of thrombospondin-1

**DOI:** 10.1186/s12881-020-01118-7

**Published:** 2020-09-11

**Authors:** Urban Alehagen, Levar Shamoun, Dick Wågsäter

**Affiliations:** 1 Institution of Medical and Health Sciences, Division of Cardiovascular Medicine, Department of Medicine and Health Sciences, Faculty of Health Sciences, Linköping University, SE-581 85 Linköping, Sweden; 2 Division of Medical Diagnostics, Department of Laboratory Medicine, Jönköping County, Jönköping, Sweden; 3 Department of Medical Cell Biology, Uppsala University, Uppsala, Sweden

**Keywords:** TSP-1, Genotypes, Elderly, Genders, Mortality

## Abstract

**Background:**

Cardiovascular diseases are still the major cause of death in the Western world, with different outcomes between the two genders. Efforts to identify those at risk are therefore given priority in the handling of health resources. Thrombospondins (TSP) are extracellular matrix proteins associated with cardiovascular diseases. The aim of this study was to investigate variations in single nucleotide polymorphisms (SNPs) of TSP-1 and plasma expression, and associations with mortality from a gender perspective.

**Methods:**

A population of 470 community-living persons were invited to participate. The participants were followed for 7.9 years and underwent a clinical examination and blood sampling. SNP analyses of TSP-1 rs1478604 and rs2228262 using allelic discrimination and plasma measurement of TSP-1 using ELISA were performed,

**Results:**

During the follow-up period, 135 (28.7%) all-cause and 83 (17.7%) cardiovascular deaths were registered.

In the female population, the A/A genotype of rs2228262 and the T/T genotype of rs1478604 exhibited significantly more cardiovascular deaths compared with the A/G and G/G, or the T/C and C/C genotypes amalgamated (rs2228262: 13.7% vs 2.0%; Χ^2^:5.29; *P* = 0.02; rs1478604:17.7% vs 4.7%; Χ^2^:9.50; *P* = 0.002). Applied in a risk evaluation, the A/A, or T/T genotypes exhibited an increased risk of cardiovascular mortality (rs2228262: HR: 7.1; 95%CI 1.11–45.8; *P* = 0.04; rs1478604: HR: 3.18; 95%CI 1.35–7.50; *p* = 0.008). No differences among the three genotypes could be seen in the male group.

**Conclusion:**

In this study the female group having the A/A genotype of rs2228262, or the T/T genotype of rs1478604 of TSP-1 exhibited higher cardiovascular mortality after a follow-up of almost 8 years. No corresponding genotype differences could be found in the male group. Genotype evaluations should be considered as one of the options to identify individuals at risk. However, this study should be regarded as hypothesis-generating, and more research in the field is needed.

## Background

As cardiovascular diseases are a major cause of deaths in the Western world [1], the economic consequences of missed treatment are high. Cardiovascular diseases represented almost 30% of all deaths in a prospective multinational cohort of more than 155,000 individuals [2] . One of the strategies to minimise the health expenditures and to improve health-related quality of life for the patient could be to identify patients at risk.

The thrombospondin group consists of five different glycoproteins [3]. Among these Thrombospondin-1 (TSP-1) is an extracellular matrix protein that is expressed by various cell types where it can modulate angiogenesis [4], cell proliferation and migration [5] during vascular remodelling. Also, TSP-1 is upregulated in pulmonary hypertension, and is believed to promote vasculopathy and dysfunction [6]. Interesting data have been reported indicating an association between a low level of TSP-1 after percutaneous coronary intervention and risk of major adverse cardiac events [7]. An early study by Topol and colleagues found that single nucleotide polymorphisms (SNP) in TSP genes were associated with myocardial infarction [8] . However, validation studies could not support this [9].

Therefore, this study had two aims, namely 1. To explore possible associations of SNPs of rs1478604 and rs2228262, and 2. To determine if high plasma levels of TSP-1 were associated with higher mortality in an elderly group of community-living persons during a follow-up period of more than 7 years.

## Methods

### Patient population

The methods and the project evaluations have previously been described [10, 11]. Thus, in this sub-study 239 males, and 231 females with a mean age of 77.0 years (range: 18 years), living in south-east of Sweden were included. They were all part of a longitudinal epidemiological study with a focus on cardiovascular risk factors.

In order to minimise the problem of selection bias, all participants in a specific age, and living in the specific area were invited to participate in the sub-study that was conducted between 2003 and 2005. Besides a new patient history, blood samples were delivered, and all participants were submitted to an electrocardiogram (ECG) and echocardiographic examination. In order to classify the physical performance of the participants, the New York Heart Association functional class (NYHA class) classification was applied. In this classification no limitation of activity renders class I, whereas symptoms already at rest renders class IV [12]. The classification was evaluated by the including physician based on the patient history.

The study protocol was approved by the Regional Ethical Review Board of Linköping, Sweden (Dnr 95,044), and all participants gave their written informed consent, and the study was conducted in accordance with the Declaration of Helsinki principles.

All mortality information was based on autopsy reports or from the National Board of Health and Welfare in Sweden, which registers all deaths.

### Co-morbidity

In this sub-study hypertension (HT) has been defined as a blood pressure > 140/90 mmHg as measured in the right arm, and with the participant in a supine position, measured after 30 min of rest, or if the participant already had received the diagnosis.

The diagnosis diabetes was assumed if the participant already had received the diagnosis, or had a fasting blood glucose ≥7 mmol/L.

Ischemic heart disease (IHD) was defined if the participant had a previous history of angina pectoris or a previous myocardial infarction, or signs of a previous myocardial infarction on ECG.

Heart failure was defined if the participant had already received the diagnosis, or symptoms and/or signs of heart failure and objective signs of reduced cardiac function as seen on echocardiography.

Cardiovascular mortality was defined if the cause was fatal arrythmias, myocardial infarction, heart failure, or cerebrovascular insult.

### Echocardiographic examination

All echocardiographic examinations have been performed using an Accuson XP-128P, and with the participant in a supine, and left position on the bench. Only the systolic cardiac function has been examined. Normal cardiac systolic function was defined as EF ≥ 50%, and severely impaired cardiac systolic function as EF < 30%.

### Determination of TSP-1 expression in plasma

All blood samples were obtained at the start of the study, while the patients were at rest in a supine position, and all samples were collected in pre-chilled plastic Vacutainer tubes (Terumo EDTA K-3). Plasma was prepared by centrifugation at 3000 g for 10 min at 4 °C. All samples were stored at -70 °C until analysis. None of the samples were thawed more than twice. ELISA (# DTSP10, Bio-Techne, RnD systems, USA) was used to assess TSP-1 expression in plasma. In brief, levels were determined from a standard curve with absorbance read at 450 nm with wave-length correction at 540 nm.

### Genotype determination

Genomic DNA was isolated from peripheral blood using the QIAmp DNA Mini Kit (QIAGEN, Germany), following the manufacturer’s instructions. DNA (10 ng) was analysed with NanoDrop and was mixed with Taqman genotyping master mix (Thermo Fisher Scientific, Applied Biosystems, Sweden) and was amplified using the 7500 Fast Real-Time PCR system (Applied Biosystems). Genotypes of SNPs rs2228262 (# C__16170900_10) and rs1478604 (# C___3100547_20) were analysed with the 7500 Fast Real-Time PCR system with allelic discrimination using TaqMan SNP probes (Applied Biosystems) according to a previous protocol [10]. The two SNPs, belonging to two different haploblocks, one in the 5’UTR and one in one of the exons, were selected from relevant results in the literature. Amplification was performed using an initial cycle at 50 °C for two minutes, followed by one cycle at 95 °C for 10 min, and finally 40 cycles at 95 °C for 15 s and at 60 °C for one minute. Internal controls and negative controls were included in all PCRs. The manual calling option in the allelic discrimination application ABI PRISM 7500 SDS software, version 1.3.1 (Applied Biosystems) was used to assign genotypes. A total call rate of 98.5% was achieved.

### Statistical methods

The statistical methods used in this sub-study are mainly the same as used previous publications [10]. Descriptive data are presented as percentages or mean and standard deviation (SD). Hypothesis testing were performed using the student’s unpaired two-sided T-test, and the chi-square test was applied for discrete variables. Cox proportional hazard regression analyses (both univariate and multivariate) were used to evaluate the risk of mortality during the follow-up period. Both all-cause- and cardiovascular mortality were analysed. Kaplan-Meier graphs were used to illustrate cardiovascular mortality during the follow-up time. Censored patients were those who were still alive at the end of the study period or who had died of causes other than cardiovascular disease. Completed patients were who had died a cardiovascular death. In the multivariate multivariable regression models, adjustments were made for the following co-variates; age, hypertension, diabetes, IHD, atrial fibrillation, EF < 40%, Hb < 120 g/L, ACE-inhibitors/Angiotensin receptor blockers, beta-blockers, and diuretics.

In the Cox regressions, the G/G and A/G genotypes of rs2228262 or the C/C and T/C genotypes of rs1478604 were amalgamated because of the small group sizes of the G/G genotype of rs2228262 (0.9% of the total population), and of the C/C genotype of the rs1478604 genotype, and because the two amalgamated genotypes had approximately the same CV mortality in the rs1478604 genotype (C/C: 13.8% vs T/C: 13.7%). Regarding the rs2228262 genotype, the A/G genotype had a CV mortality of 14.0% whereas the G/G genotype was so small that no CV mortality was observed during the follow-up-period.

When evaluating the distribution of the plasma levels of TSP-1 in the different genotypes of the two SNPs, ANOVA evaluation was used.

A *P*-value < 0.05 was considered statistically significant. All data were analysed using standard software packages (Statistica v. 13.2, Statsoft Inc., Tulsa, OK, USA).

## Results

The basal characteristics of the study population are presented in Table 1. Almost equal numbers of males and females were included (239 vs. 231), and the two genders had the same mean age, 77 years. A preponderance of males with IHD was seen; 66/239 (27.6) versus 44/231 (19.0) in the female group, as seen in other elderly populations [13–15]. The proportions treated with beta-blockers or ACEI/AII were equal between the two genders. However, a preponderance of patients on treatment with diuretics in the female group 95/231(41.1), vs. 77/239 (32.2) in the male group could be seen, as in many other elderly populations [16–22]. A greater proportion of the population with anaemia was found in the female population, 33/231(14.3) vs. 18/239 (7.5) in the male population. Also, as could be expected, a greater proportion of the population with impaired systolic cardiac function could be found in the male group, 27/239 (11.3), vs. 9/231(3.9) in the female group, a finding that is not surprising taking into consideration the preponderance of myocardial infarctions among males as compared with females in middle age [23, 24].

**Table 1:** Basal characteristics of the study population

Variable	Males	Females	*p*-values
n	239	231	
Age, years (SD)	77 (3)	77 (4)	0.61
History			
Diabetes, n (%)	85 (35.6)	68 (29.4)	0.16
Hypertension, n (%)	173 (72.4)	184 (79.7)	Χ^2^:4.28 *P* = 0.04
IHD, n (%)	66 (27.6)	44 (19.0)	Χ^2^:4.81 *P* = 0.03
Atrial fibrillation, n (%)	28 (11.7)	18 (7.8)	0.15
NYHA I, n (%)	117 (49.0)	121 (52.4)	0.46
NYHA II, n (%)	73 (30.5)	64 (27.7)	0.50
NYHA III, n (%)	48 (20.1)	46 (19.9)	0.96
NYHA IV, n (%)	0	0	
Medication			
Beta blockers, n (%)	90 (37.7)	78 (33.8)	0.38
ACEI/ARB, n (%)	62 (25.9)	60 (26.0)	0.99
Diuretics, n (%)	77 (32.2)	95 (41.1)	Χ^2^:4.02 *P* = 0.05
Examinations			
BP systolic, mm Hg, mean (SD)	146 (23)	151 (19)	*P* = 0.34
BP diastolic, mmHg, mean (SD)	75 (12)	75 (10)	0.80
Hb < 120 g/L, n (%)	18 (7.5)	33 (14.3)	Χ^2^:5.54 P = 0.02
EF < 40%, n (%)	27 (11.3)	9 (3.9)	Χ^2^:9.10 *P* = 0.003

*ACEI* angiotensin converting enzyme inhibitors, *ARB* angiotensin receptor blockers, *BP* blood pressure, *EF* ejection fraction, *IHD* ischemic heart disease, *NYHA* New York Heart Association functional class, *SD* standard deviation

Regarding the SNP rs1478604, results were obtained from 470 individuals, whereas from the SNP rs2228262, results were obtained from 466 individuals, a difference due to the call rate of the PCR (98.5%).

The distribution of the three genotypes in the two genders is presented in Tables 2 and 3 with no significant differences between the genders, and where the A/A, or T/T genotypes represented the most common genotypes in both SNPs.

**Table 2:** Distribution of the three genotypes of rs2228262 in the study population

Genotype	Males	Females	*P*-value
A/A, n (%)	186/235 (79.1)	181/231 (78.4)	0.83
A/G, n (%)	48/235 (20.4)	45/231 (19.5)	0.80
G/G, n (%)	1/235 (0.4)	3/231 (1.3)	–

**Table 3:** Distribution of the three genotypes of rs1478604 in the study population

Genotype	Males	Females	*P*-value
T/T, n (%)	135/239 (56.5)	124/231 (53.7)	0.54
T/C, n (%)	92/239 (38.5)	90/231 (39.0)	0.92
C/C, n (%)	12/239 (5.0)	17/231 (7.4)	0.29

### TSP-1 levels in plasma

In the study population determination of expression of TSP-1 in plasma from 470 individuals was performed, and the levels were compared in different subgroups (Table 4); IHD, hypertension, diabetes and in the male group. Only in those with hypertension could a significant difference be seen, with a higher proportion of those with a plasma level in the fourth quartile. In no other subgroup could any difference be demonstrated. The mean plasma concentration for TSP-1 in the study population was 1401 ng/nL (SD: 1583 ng/mL). The distributions of plasma concentration in the total study population and in the two genders are presented in Table 5.

**Table 4:** Relation of different variables in the 1st and the 4th quartile of plasma concentration of TSP1 in the study population

Variable	Q1 (< 756 ng/mL)	Q4 (> 1600 ng/mL)	*p*-value
CV mortality, n (%)	17/117 (14.5)	23/117 (19.7)	*P* = 0.33
IHD, n (%)	21/117 (17.9)	31/117 (26.5)	*P* = 0.12
HT, n (%)	84/117 (71.8)	99/117 (84.6)	Χ^2^: 5.64; *p* = 0.018
DM, n (%)	36/117 (30.8)	48/117 (41.6)	*P* = 0.10
Male gender, n (%)	53/117 (48.7)	60/117 (51.3)	*P* = 0.69

*CV* cardiovascular, *DM* diabetes, *HT* hypertension, *IHD* ischemic heart disease

**Table 5:** Plasma concentration of TSP-1 distributed in the total population, and in the two genders

	Total population	Male group	Female group	***P***-value
Plasma concentration, median, ng/mL	1115	1130	1079	0.96
IQR	845	829	854	

**Fig. 1:**
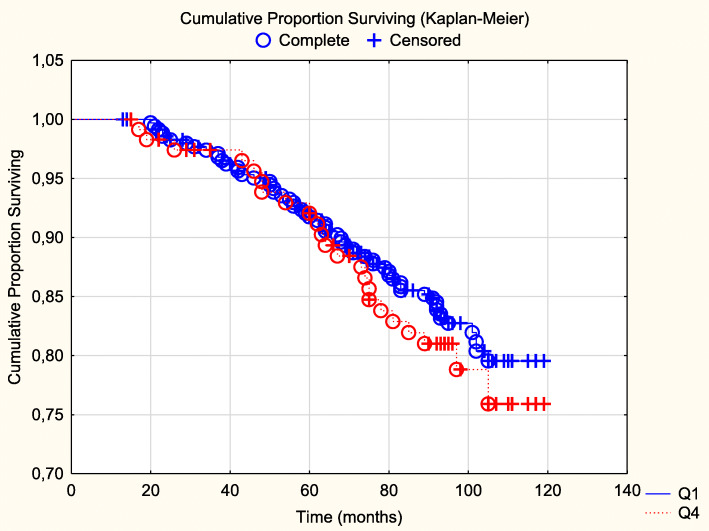
Cardiovascular mortality in those with the first quartile versus those with the highest quartile of TSP-1 in plasma during a follow-up of 7.9 years. Note: Censored participants were those still living at the end of the study period, or who had died for reasons other than cardiovascular disease. Completed participants were those who had died due to cardiovascular disease

An evaluation of CV mortality in the first versus the fourth quartiles of expression of TSP-1 was also performed (Fig. 1). No difference in CV mortality between the two groups could be found (Z = 0.65; *P* = 0.52).

The mean plasma concentration of TSP-1 was evaluated in the different SNPs in rs2228262 and rs1478604 of TSP-1. No difference in plasma concentration could be found between the SNPs of the two genotypes (Suppl. Table 1).

### Mortality and genotypes

The median follow-up time of the population was 95 months (7.9 years), and during that time 135 (28.7%) all-cause and 83 (17.7%) cardiovascular deaths were registered. Analysing the three genotypes it could be demonstrated that the A/A genotype (rs2228262) or the T/T genotype (rs1478604) was the most prevalent in both SNPs (rs2228262: 79.1%; rs1478604: 55.1%), whereas the G/G genotype (rs2228262) or the C/C genotype (rs1478604)) was the least common (rs2228262: 0.9%; rs1478604: 6.2%). The distribution of the three genotypes was mainly in concurrence with other reports [25].

#### Rs2228262

**Fig. 2:**
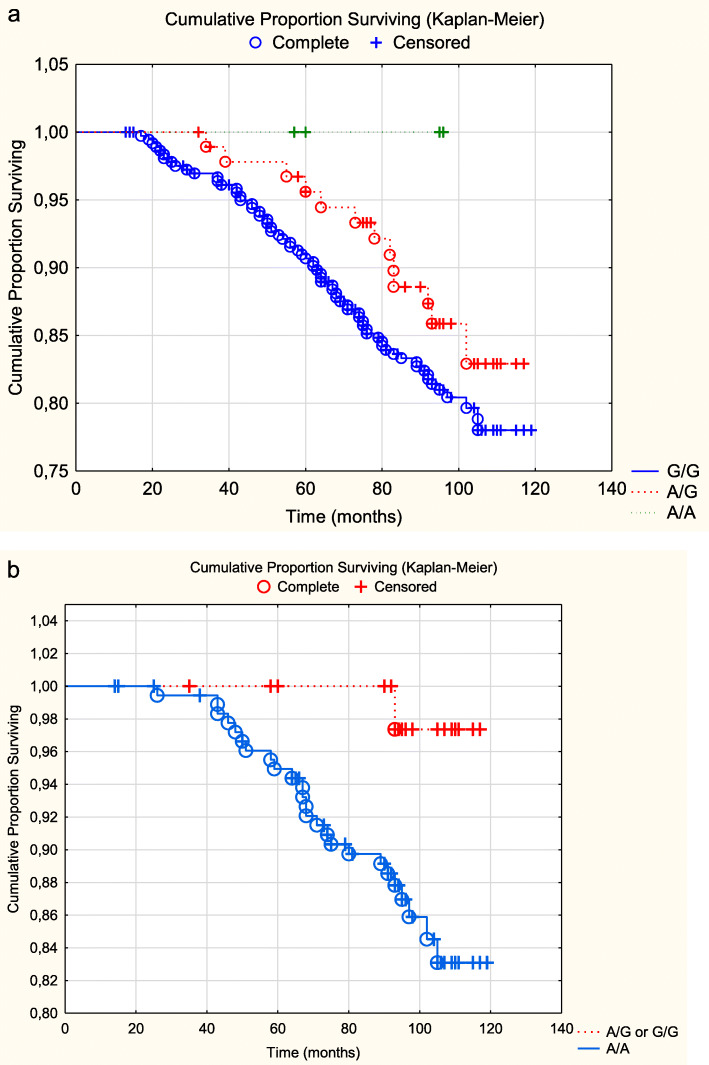
**a** Cardiovascular mortality of the three genotypes of rs2228262 in the total study population during a follow-up time of 7.9 years. Note: Censored participants were those still living at the end of the study period, or who had died for reasons other than cardiovascular disease. Completed participants were those who had died due to cardiovascular disease. **b**. Cardiovascular mortality of the three genotypes of rs2228262 in the female population during a follow-up time of 7.9 years. Note: Censored participants were those still living at the end of the study period, or who had died for reasons other than cardiovascular disease. Completed participants were those who had died due to cardiovascular disease

In the SNP rs2228262 a cardiovascular mortality in the A/A genotype of 69/367 (18.8%) could be found, as compared to 13/97 (13.4%) in the A/G or G/G amalgamated group, thus there was no significant difference (Χ^2^:1.54; *P* = 0.22). If evaluating all three genotypes of the SNP regarding CV mortality, still an insignificant difference was obtained as can be seen from the Kaplan-Meier graph (Fig. 2a). Regarding all-cause mortality; no significant difference could be found between the A/A genotype (108/367; 29.4%), versus the A/G – G/G group (24/97; 24.7%; (χ^2^ = 0.83; *P* = 0.36).

However, analysing the females, a cardiovascular mortality of the A/A group could be seen in 25/181 (13.6%), while for the amalgamated A/G or G/G group the ratio was 1/48 (2.1%) cases, thus there was a significant difference (Χ^2^:5.29; *P* = 0.02). This can also be seen in the Kaplan-Meier graphs, where we chose to illustrate all three genotypes (Fig. 2b). Regarding all-cause mortality in the female group, the ratios were 39/181 (21.5%) in the A/A group, and 5/48 (10.4%) in the A/G or G/G groups amalgamated, thus there was not a significant difference (Χ^2^:3.15; *P* = 0.08).

Analysing the male group, a cardiovascular mortality in the A/A group could be seen in 44/186 (23.7%) vs. 12/49 (24.5%) in the A/G or G/G amalgamated group, thus there was no significant difference; χ^2^ = 0.01; *P* = 0.93). Regarding all-cause mortality in the male group, no significant difference could be seen between the two groups (A/A: 69/186; 37.1% vs A/G or G/G: 19/49; 38.8%; χ^2^ = 0.03; *P* = 0.87).

We also applied risk evaluation for cardiovascular death as calculated in a multivariate risk model. From that, it could be found that females with the A/A genotype showed an increased risk for cardiovascular mortality. The point estimate was high (HR:7.12); however, the confidence interval was wide (1.11–45.78), so the results should be interpreted with caution, even though a significant increased risk could be seen (Table 6). Regarding all-cause mortality, no significant increased risk could be seen. In the male group, no significant differences in risk could be found between the genotypes.

**Table 6:** Cox proportional hazard regression analysis evaluating risk of cardiovascular mortality in the study population divided in the total population, and into the two genders regarding rs2228262 of TSP-1

Variable		Total population			Females			Males	
	HR	95%CI	p-value	HR	95%CI	***p***-value	HR	95%CI	***p***-value
Age	1.18	1.11–1.26	< 0.0001	1.17	1.05–1.31	0.005	1.22	1.12–1.32	< 0.0001
IHD	1.33	0.79–2.24	0.29	1.85	0.70–4.88	0.21	0.93	0.48–1.82	0.84
Hypertension	1.26	0.74–2.16	0.40	2.10	0.75–5.86	0.16	1.23	0.66–2.29	0.52
Diabetes	1.21	0.76–1.92	0.41	2.06	0.90–4.73	0.09	0.84	0.48–1.48	0.55
AECI/ARB	1.03	0.63–1.70	0.91	0.65	0.26–1.63	0.36	1.29	0.70–2.38	0.41
Beta blockers	0.75	0.46–1.24	0.26	0.61	0.27–1.42	0.25	0.73	0.38–1.39	0.33
Atrial fibr.	2.02	1.11–3.66	0.02	4.72	1.60–13.92	0.005	1.83	0.91–3.67	0.09
Hb < 120 g/L	1.55	0.86–2.79	0.14	3.70	1.52–9.00	0.004	1.10	0.48–2.54	0.82
EF < 40%	1.77	0.93–3.37	0.08	3.12	0.82–11.85	0.09	1.62	0.72–3.66	0.24
TSP-1 rs2228262, A/A	1.48	0.80–2.75	0.21	7.12	1.11–45.78	0.04	1.14	0.59–2.23	0.69

*ACEI* angiotensin convering enzyme inhibitors, *ARB* angiotensin receptor inhibitors, *EF* ejection fraction, *HR* hazard ratio

#### Rs1478604

In those with the T/T genotype a cardiovascular mortality of 54/259 (20.8%) could be found, and in the amalgamated group consisting of the genotypes T/C and C/C, a cardiovascular mortality of 29/211 (13.7%) was found, thus there was a significantly higher mortality in the T/T group (Χ^2^:4.04; *P* = 0.045). Regarding all-cause mortality; in the T/T group the ratios were 79/180 (30.5%) versus the amalgamated T/C and C/C groups 56/211 (26.5%) (Χ^2^:3.34; *P* = 0.07), thus a trend could be observed, but not a significant difference.

Analysing the females, a cardiovascular mortality in the T/T group could be seen in 22/124 (17.7%) whereas in the amalgamated T/C or C/C group the ratio was 5/107 (4.7%) cases, thus there was a significant difference (Χ^2^:9.50; *P* = 0.002). The corresponding figures of all-cause mortality in the female group were 25/124 (20.2%) in the T/T group, and 17/107 (15.9%) in the T/C or C/C groups amalgamated, thus there was not a significant difference (χ^2^ = 0.71; *P* = 0.40).

In the male group, a cardiovascular mortality in the T/T group of 34/135 (25.1%) vs 22/104 (21.2%) in the T/C or C/C groups could be seen, which was not a significant difference (χ^2^ = 0.53; *P* = 0.47). Regarding all-cause mortality in the male group, no significant difference could be seen between the two groups (T/T: 52/135; 38.5% vs T/C or C/C: 37/104; 35.6%; χ^2^ = 0.22; *P* = 0.64).

Applying the mortality data into a multivariate model including the most well-known cardiovascular risk factors, the T/T genotype displayed a significant increased cardiovascular risk (HR:1.62; 95%CI 1.03–2.53; *P* = 0.04), (Table 7). Moreover, an increased cardiovascular risk could only be found in the female group (HR: 3.18; 95%CI 1.35–7.50; *P* = 0.008), whereas in the male group no increased risk could be seen in the T/T genotype (HR: 1.18; 95%CI 0.68–2.05).

**Table 7:** Cox proportional hazard regression analysis evaluating risk of cardiovascular mortality regarding rs1478604 of TSP-1 in the study population divided in the total population, and into the two genders

Variable		Total population			Females			Males	
	HR	95%CI	***p***-value	HR	95%CI	***p***-value	HR	95%CI	***p***-value
Age	1.17	1.10–1.25	< 0.0001	1.20	1.07–1.34	0.002	1.20	1.10–1.30	< 0.0001
IHD	1.23	0.74–2.03	0.42	1.13	0.44–2.89	0.80	1.05	0.55–2.01	0.88
Hypertension	1.17	0.71–1.93	0.53	1.85	0.72–4.71	0.20	1.04	0.58–1.89	0.89
Diabetes	1.25	0.82–1.93	0.30	1.90	0.86–4.18	0.11	0.87	0.50–1.50	0.61
AECI/ARB	1.00	0.62–1.61	0.99	0.73	0.32–1.66	0.45	1.30	0.72–2.36	0.39
Beta blockers	0.77	0.48–1.24	0.28	0.76	0.35–1.65	0.48	0.78	0.42–1.46	0.43
Atrial fibr.	2.23	1.30–3.86	0.004	4.66	1.69–12.83	0.003	1.71	0.85–3.45	0.13
Hb < 120 g/L	1.42	0.82–2.49	0.21	2.52	1.07–5.94	0.04	1.03	0.44–2.40	0.94
EF < 40%	1.79	0.96–3.33	0.07	3.57	1.14–11.20	0.03	1.41	0.63–3.16	0.40
TSP-1 rs1478604, T/T	1.62	1.03–2.53	0.04	3.18	1.35–7.50	0.008	1.18	0.68–2.05	0.56

*ACEI* angiotensin convering enzyme inhibitors, *ARB* angiotensin receptor inhibitors, *EF* ejection fraction, *HR* hazard ratio

In an effort to evaluate a possible skewness regarding cardiovascular risk factors as a possible explanation for the gender differences obtained between the T/T and the T/C or C/C groups, the relation of risk factors is presented in Table 8 for rs2228262, and in Table 9 for rs1478604. No difference between the groups in relation to cardiovascular risk factors could be seen.

**Table 8:** Distribution of different well-known risk factors for cardiovascular mortality between the genotypes A/A and A/G or G/A amalgamated, of rs2228262 in the study population

Variable		Total pop			Females			Males	
	A/A	A/G-G/G	***p***-value	A/A	A/G-G/G	***p***-value	A/A	A/G-G/G	***p***-value
n	259	211		124	107		135	104	
IHD,n (%)	65 (25.1)	45 (21.3)	0.34	23 (18.5)	21 (19.6)	0.84	42 (31.1)	24 (23.1)	0.17
DM, n (%)	90 (34.7)	63 (29.9)	0.26	41 (33.1)	27 (25.2)	0.19	49 (36.3)	36 (34.6)	0.79
NYHA III, n (%)	57 (22.0)	37 (17.5)	0.23	27 (21.8)	19 (17.8)	0.45	30 (22.2)	18 (17.3)	0.35
AF, n (%)	31 (12.0)	15 (7.1)	0.08	12 (9.7)	6 (5.6)	0.25	19 (14.1)	9 (8.7)	0.20
EF < 40%, n (%)	19 (7.3)	17 (8.1)	0.77	6 (4.8)	3 (2.8)	0.43	13 (9.6)	14 (13.5)	0.35
Hb < 120 g/L, n (%)	25 (9.7)	26 (12.3)	0.35	17 (13.7)	16 (15.0)	0.79	8 (2.2)	10 (9.6)	0.28

*AF* atrial fibrillation, *DM* diabetes mellitus, *EF* ejection fraction, *IHD* ischemic heart disease, *NYHA* New York Heart Association functional class

**Table 9:** Distribution of different well-known risk factors for cardiovascular mortality between the genotypes T/T and T/C or C/C amalgamated, of rs1478604 in the study population

Variable		Total pop			Females			Males	
	T/T	T/C-C/C	***p***-value	T/T	T/C-C/C	***p***-value	T/T	T/C-C/C	***p***-value
n	259	211		124	107		135	104	
IHD,n (%)	65 (25.1)	45 (21.3)	0.34	23 (18.5)	21 (19.6)	0.84	42 (31.1)	24 (23.1)	0.17
DM, n (%)	90 (34.7)	63 (29.9)	0.26	41 (33.1)	27 (25.2)	0.19	49 (36.3)	36 (34.6)	0.79
NYHA III, n (%)	57 (22.0)	37 (17.5)	0.23	27 (21.8)	19 (17.8)	0.45	30 (22.2)	18 (17.3)	0.35
AF, n (%)	31 (12.0)	15 (7.1)	0.08	12 (9.7)	6 (5.6)	0.25	19 (14.1)	9 (8.7)	0.20
EF < 40%, n (%)	19 (7.3)	17 (8.1)	0.77	6 (4.8)	3 (2.8)	0.43	13 (9.6)	14 (13.5)	0.35
Hb < 120 g/L, n (%)	25 (9.7)	26 (12.3)	0.35	17 (13.7)	16 (15.0)	0.79	8 (2.2)	10 (9.6)	0.28

*AF* atrial fibrillation, *DM* diabetes mellitus, *EF* ejection fraction, *IHD* ischemic heart disease, *NYHA* New York Heart Association functional class

**Fig. 3:**
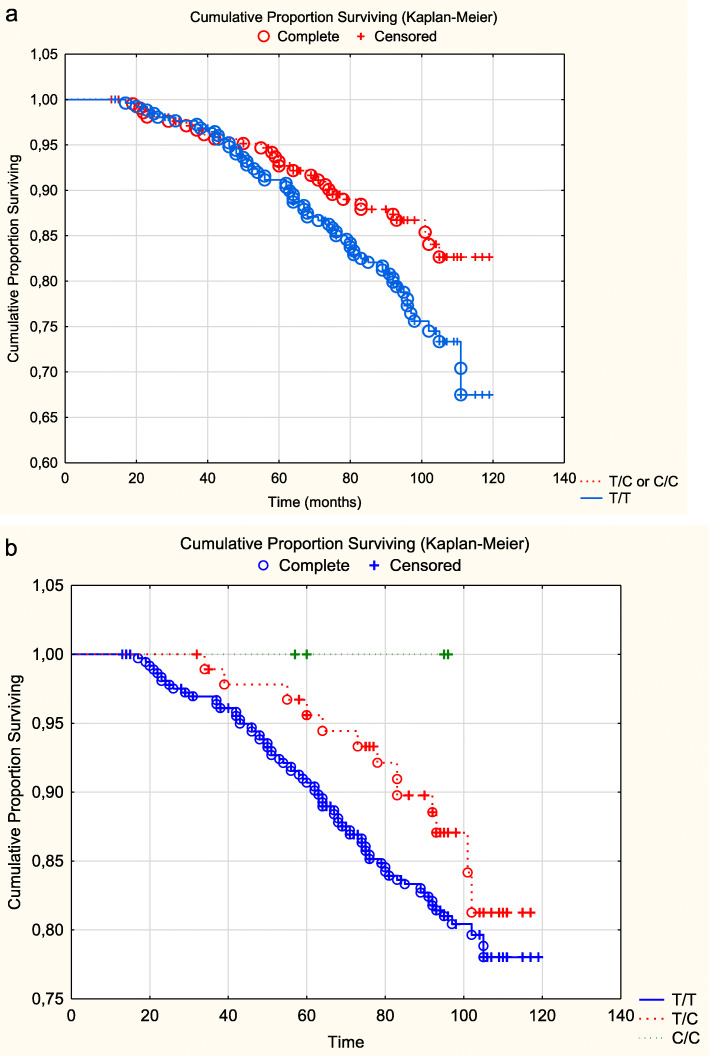
**a** Cardiovascular mortality of the three genotypes of rs1478604 in the total study population during a follow-up time of 7.9 years. Note: Censored participants were those still living at the end of the study period, or who had died for reasons other than cardiovascular disease. Completed participants were those who had died due to cardiovascular disease. **b**. Cardiovascular mortality of the three genotypes of rs1478604 in the total female population during a follow-up time of 7.9 years. Note: Censored participants were those still living at the end of the study period, or who had died for reasons other than cardiovascular disease. Completed participants were those who had died due to cardiovascular disease

In a survival analysis of the total study population, a significant difference in survival could be demonstrated between the two groups, T/T versus T/C or C/C, regarding cardiovascular mortality (z = 2.09; *P* = 0.04) (Fig. 3a).

Evaluating the female group, an even greater difference between the two groups could be found (z = 2.34; *P* = 0.02) (Fig. 3b). In the male population, no significant difference in cardiovascular mortality between the two groups could be found (z = 0.91; *P* = 0.36).

## Discussion

TSP-1 has a multitude of actions in different processes in the body, including angiogenesis, inflammation and cardiac fibrosis [26, 27]. This evaluation aimed to explore whether certain genotypes of the rs2228262 and rs1478604 of TSP-1 could be identified as associated with a higher risk for mortality, as increased focus and research on potential genetic polymorphisms in specific diseases have developed in recent years. Also, in clinical medicine, reports have demonstrated an association between the level of TSP-1 and prognosis in lung cancer [28], and high plasma levels of TSP-1 have been reported to correlate positively with cardiovascular disease [29]. That the different genotypes of TSP-1 could have a possible influence on mortality is logical, as thrombospondin itself is involved in the different processes that are fundamental to the progress of atherosclerosis, of which inflammation is one of the most important [30]. In this evaluation, we could, in a small hypothesis-generating study, demonstrate that the A/A genotype of the SNPs rs2228262 and T/T genotype of rs1478604, showed a significantly higher risk for cardiovascular mortality, and a trend towards, rather than higher risk of all-cause mortality. The trend value could be partly explained by the small sample size. Rs2228262 (A > G) is located in amino acid position 700 in exon 13 and results in a change in asparagine to serine residue in a domain coding for calcium binding [31–33]. An alteration in the calcium binding of the heart, vessels and platelets could cause several potential physiological effects resulting in cardiovascular mortality. Rs1478604 (T > C) is located within the 5-untranslated region (UTR) with possible influence of the translational regulation. As an example, there is a decreased expression of TSP1 mRNA in ocular surface epithelia derived from individuals genotyped with C genotype [34]. However, we could not find any association between the T or C genotype of rs1478604 and plasma expression in our study.

That the T/T genotype might have a different influence on immunological mechanisms, compared to the two other genotypes is illustrated in a report on corneal allograft rejection, where the T/T genotype showed a more than 50% increased risk for rejection, compared to the two other genotypes [35].

In the literature only a few reports on the rs2228286 genotype could be found. However, Koch et al. published a small meta-analysis and a case-control study of different genotypes of TSP-1, including rs2228262 [9]. It should be noted that the focus for that study was to evaluate the association between the genotype and myocardial infarction, and thus both the myocardial infarction group and the control group were evaluated with coronary angiograms, thus a clear indication was present also for the control group. The authors concluded that no significant association between rs2228262 and myocardial infarction could be seen. However, the fact that the control group could not be regarded as “healthy” may have influenced the result. In the presented evaluation we have also applied a follow-up time of nearly 8 years, which make the results interesting, even if the sample sizes were small.

That TSP-1 has an association with tumour development has been reported by Sun et al., presenting data from 24 studies covering almost 2400 patients [36]. They reported that high expression of TSP-1 indicated a poor prognosis of breast cancer and gynaecological cancer. As already mentioned, TSP-1 has a multitude of actions, including tissue repair, inhibition of angiogenesis, and even anti-tumour activity [37]. Thus, an influence on the vascular properties resulting in cardiovascular effects is not surprising.

### Gender differences

In the female population, the individuals with the A/A genotype of rs2228262 or the T/T genotype of rs1478604 of TSP-1 had significantly higher cardiovascular mortality compared to the amalgamated A/G and G/G (rs2228262) or the T/C and C/C groups (rs1478604) (rs2228262: 13.7% vs 2.0%; rs1478604: 17.7% vs 4.7%) whereas a corresponding difference in mortality among the male individuals could not be found (rs2228262: 23.4% vs 25.0%; rs1478604: 30.7% vs 26.8%). In order to explore whether the differences between the A/A or T/T genotypes and the A/G-G/G or T/C-C/C groups could be explained by other factors, the distribution of some well-known risk factors for cardiovascular mortality have been presented. No preponderance of any of the evaluated risk factors could be seen in either the A/A (T/T), or the A/G-G/G (T/C-C/C) groups, or in the total population, or in either of the two genders. Therefore, the explanation of the difference in mortality between the two groups does not seem to be based on confounding, but instead on a possible specific genotype difference, as no differences in mortality could be demonstrated between those with a high expression of TSP-1 versus those with a low expression. However, this specific mechanism for the difference is presently unknown. Genetic differences with regard to cardiovascular risk in other biomarkers have also been reported by our group [10].

### Clinical implications

The presented genotype difference of the two SNPs between the two genders is of clinical importance as it is of interest to identify those at high risk. Even if it is not possible to modify the genetic constitution, it is possible to implement more intense prevention procedures and an individualised follow-up programme for those patients, which also may reduce CV disease and mortality and thus health expenditure for society.

On the other hand, the females with the A/G or G/G genotypes exhibited a cardiovascular mortality of only 4.7% after almost 8 years in this elderly population, and this gives an important message at a time with restricted health resources.

For the clinician who, in everyday practice, is faced with patients with symptoms of cardiovascular disease, a genetic evaluation of specific candidate genes/ genotypes could help in providing a personal and optimal follow-up programme after performing a guideline-directed investigation followed by treatment.

### Limitations

The above presentation is a community-based study, including all persons in a specified age stratum, and therefore the majority has no symptoms from the heart, Therefore the group with cardiovascular disease are in a minority and thus the size of that group is small, resulting in wide confidence intervals in risk evaluations, thus making their interpretation uncertain. However, it could be argued that a message regarding cardiovascular risk can still be found.

The evaluated population study population is an elderly one, and extrapolating the results into another age group should be done with caution..

Moreover, the groups with certain genotypes are small; therefore, the results of some of the evaluations should be interpreted with caution, and the results should be regarded as hypothesis-generating.

## Conclusion

An evaluation of the A/A, A/G and G/G genotypes of the SNPs rs2228262 and the genotypes T/T, T/C and C/C of rs1478604 and in TSP-1 is reported here. By applying a follow-up period of almost 8 years, a significantly increased cardiovascular mortality could be seen in the A/A genotype (rs2228262) and the T/T genotype of rs1478604, compared with the A/G or G/G genotypes (rs2228262) or the T/C or C/C genotypes of rs1478604. The difference in cardiovascular mortality was mainly driven by the result from the female group. Applying the results into risk evaluation, the A/A group exhibited a 3.18-fold increased risk of cardiovascular mortality among the females. In the male group however, no such gender difference could be found.

As the study is small it should be regarded as hypothesis-generating, and therefore more research in the area is proposed in order to determine whether the genotype evaluation could also be launched in clinical practice.

## Data Availability

Under Swedish Law, the authors cannot share the data underlying this study and cannot do any further research than what is specified in the ethical permissions application. For inquires on the data, researchers should first reach out to the owner of the database, the University of Linköping. Please reach out to the corresponding author with requests and for assistance with data requests. If the university approves the request, researchers can submit an application to the Regional Ethical Review Board for the specific research question that the researcher wants to examine.

## Abbreviations

CI: Confidence interval
CV: Cardiovascular
ECG: Electrocardiogram
EF: Ejection fraction
HR: Hazard ratio
SD: Standard deviation
SNP: Single nucleotide polymorphisms
TSP-1: Thrombospondin-1
